# Comparison of the EQ-5D-5L and the patient-reported outcomes measurement information system preference score (PROPr) in the United States

**DOI:** 10.1186/s41687-024-00749-1

**Published:** 2024-07-19

**Authors:** Ron D. Hays, Maria Orlando Edelen, Anthony Rodriguez, Nabeel Qureshi, David Feeny, Patricia M. Herman

**Affiliations:** 1 Division of General Internal Medicine and Health Services Research, UCLA Department of Medicine, 1100 Glendon Avenue Suite 850, Los Angeles, CA USA; 2 Patient Reported Outcomes, Value and Experience (PROVE) Center, Department of Surgery, Brigham and Women’s Hospital, Boston, MA USA; 3 Behavioral and Policy Sciences, RAND Corporation, Boston, MA USA; 4 Behavioral and Policy Sciences, RAND Corporation, Santa Monica, CA USA; 5 Department of Economics, McMaster University, Hamilton, ON Canada

**Keywords:** EQ-5D-5L, PROPr, Preference measures, PROMIS^®^, Longitudinal, Back pain

## Abstract

**Background:**

In contrast to prior research, our study presents longitudinal comparisons of the EQ-5D-5L and Patient-Reported Outcomes Measurement Information System (PROMIS) preference (PROPr) scores. This fills a gap in the literature, providing a much-needed understanding of these preference-based measures and their applications in healthcare research. Furthermore, our study provides equations to estimate one measure from the other, a tool that can significantly facilitate comparisons across studies.

**Methods:**

We administered a health survey to 4,098 KnowledgePanel^®^ members living in the United States. A subset of 1,256 (82% response rate) with back pain also completed the six-month follow-up survey. We then conducted thorough cross-sectional and longitudinal analyses of the two measures, including product-moment correlations between scores, associations with demographic variables, and health conditions. To estimate one measure from the other, we used ordinary least squares (OLS) regression with the baseline data from the general population.

**Results:**

The correlation between the EQ-5D-5L and PROPr scores was 0.69, but the intraclass correlation was only 0.34 because the PROPr had lower (less positive) mean scores on the 0 (dead) to 1 (perfect health) continuum than the EQ-5D-5L. The associations between the two preference measures and demographic variables were similar at baseline. The product-moment correlation between unstandardized beta coefficients for each preference measure regressed on 22 health conditions was 0.86, reflecting similar patterns of unique associations. Correlations of change from baseline to 6 months in the two measures with retrospective perceptions of change were similar. Adjusted variance explained in OLS regressions predicting one measure from the other was 48%. On average, the predicted values were within a half-standard deviation of the observed EQ-5D-5L and PROPr scores. The beta-binomial regression model slightly improved over the OLS model in predicting the EQ-5D-5L from the PROPr but was equivalent to the OLS model in predicting the PROPr.

**Conclusion:**

Despite substantial mean differences, the EQ-5D-5L and PROPr have similar cross-sectional and longitudinal associations with other variables. We provide the OLS regression equations for use in cost-effectiveness research and meta-analyses. Future studies are needed to compare these measures with different conditions and interventions to provide more information on their relative validity.

## Background

Health-related quality of life (HRQoL) profile measures provide information about multiple domains of physical, mental, and social health. The Patient-Reported Outcomes Measurement and Information System (PROMIS^®^)-29 is a profile measure developed with comprehensive qualitative and modern analytic methods, including item response theory [1–3]. The PROMIS-29 assesses pain intensity using a single 0–10 numeric rating item and seven health domains (physical function, fatigue, pain interference, depression, anxiety, ability to participate in social roles and activities, and sleep disturbance) using four polytomous (5 response categories) items per domain.

A preference-based score where 0 is anchored as dead and 1 as “perfect health” can be useful for comparing different therapies, such as comparative effectiveness research and economic evaluations [4]. An attempt to produce a preference scoring system using paired comparisons for the PROMIS-29 yielded implausible values (mean = 0.161 for one year on the quality-adjusted life year scale) [5]. In contrast, the standard gamble was used to estimate utilities for the PROMIS-Preference (PROPr) score [6]. The PROPr is based on item response theory estimates from six PROMIS-29 domains (physical function, pain interference, depression, fatigue, ability to participate in social roles and activities, sleep disturbance) and PROMIS cognitive function [7]. The PROPr scores can be estimated from item banks, short forms (e.g., PROMIS-29 + 2), or computer-adaptive testing. The number of possible health states is very large: 217,238,121 if look-up tables are used to estimate the IRT scores and even more if pattern-based scoring is used.

The EQ-5D-5L items refer to “Your health today” and assess mobility, self-care, usual activities, pain/discomfort, and anxiety/depression with five response options (*no problems, some problems, moderate problems, severe problems, and extreme problems*), with 3,125 possible health states [8]. The U.S. EQ-5D-5L weights were obtained using time trade-off (TTO) preference elicitation [9].

Product-moment correlations between the EQ-5D-5L and the PROPr preference-based scores of 0.61 [10], 0.70 [11], and 0.72 have been reported [12]. Mean scores were found to be significantly lower (worse scores) for the PROPr than the EQ-5D-5L, resulting in intraclass correlations between the two scores of only 0.44 [10] and 0.48 [12]. Rencz et al. [10] concluded that the EQ-5D-5L was more sensitive than the PROPr to health conditions. Hanmer et al. [11] found that the average estimated impact of 11 conditions (angina, asthma, cancer, chronic obstructive pulmonary disease, coronary artery disease, diabetes, emphysema, epilepsy, joint pain, myocardial infarction, stroke) relative to those without the condition was larger for the PROPr (−0.136) than for the EQ-5D-5L (−0.061). However, the preference values were estimated using the EQ-5D-3L crosswalk link function to the U.S. time trade-off value set rather than the EQ-5D-5L directly.

Studies comparing the EQ-5D-5L and the PROPr have been limited to cross-sectional data. For example, Klapproth et al. [13] compared the two measures in a cross-sectional study of 218 low-back pain patients at a spine center in Berlin. We extend prior cross-sectional comparisons using a large general population sample in the U.S. Pan et al. [14] noted that further work is needed to assess these preference-based measures longitudinally. This study addresses this gap by examining change over six months among those from the general population baseline sample with back pain, one of the most common types of chronic pain [15]. Prior studies have employed crosswalks to “harmonize” results across studies [16, 17]. In this study, we crosswalk the EQ-5D-5L and PROPr using regression equations in the baseline sample from the U.S. general population.

## Methods

The sample was drawn from adult members of KnowledgePanel^®^. This online panel relies on probability-based sampling methods for recruitment. It provides a representative sample of non-institutionalized adults 18 and older residing in the U.S. We administered a general health survey that included the PROMIS-29 + 2 to the full sample and a pain impact survey to the subset who reported back pain at baseline and 6-months later (see Measures below).

All surveys were administered in English. At baseline, the survey vendor (Ipsos) sent an email invitation to 7224 KnowledgePanel members on September 22, 2022, and gave them ten days to complete the general health survey. 57% (*n* = 4117) completed it. We excluded 19 who reported having one or two fake health conditions (see bona fide health conditions below) to identify careless respondents, resulting in a baseline sample of 4098 individuals. Data collection for the 6-month follow-up was from March 23 through April 15, 2023, for a subset of 1446 of those who reported back pain at baseline. Members of this subset received an email invitation to complete the follow-up survey. 82% (*n* = 1256) of the baseline respondents with back pain completed the follow-up survey.

Email reminders for the baseline and follow-up surveys were sent to non-responders on Day 3 of the field periods. Additional reminders were sent to the remaining non-responders every 3 days for up to 10 days. Respondents to the baseline survey received an entry into the KnowledgePanel sweepstakes and those with back pain who completed the pain impact survey also received a cash-equivalent incentive of $5. The same incentive was employed for the 6-month survey.

This study was approved by the RAND Human Subjects Protection Committee (2019-0651-AM02). All respondents provided electronic informed consent before starting the survey.

## Measures

### Demographic characteristics

We measured age in years, gender (female vs. male), race/ethnicity (White, Hispanic, Black, multi-racial, another race), and education: No high school diploma or general education diploma; High school graduate (high school diploma or the general educational equivalent (GED); Some college or associate degree; bachelor’s degree; master’s degree or higher.

### Health conditions

Thirteen health conditions were assessed by asking: Have you ever been told by a doctor or other health professional that you had: (1) hypertension; (2) high cholesterol; (3) heart disease; (4) angina; (5) heart attack; (6) stroke; (7) asthma; (8) cancer; (9) diabetes; (10) chronic obstructive pulmonary disease (COPD); (11) arthritis; (12) anxiety disorder; and (13) depression. In addition, the survey asked respondents if they were ever told they had “Syndomitis” (a fake condition). Further, participants were asked, “Do you currently have…” (1) allergies or sinus trouble; (2) back pain; (3) sciatica; (4) neck pain; (5) trouble seeing; (6) dermatitis; (7) stomach trouble; (8) trouble hearing; and (9) trouble sleeping. They were also asked if they currently have “Chekalism” (a fake condition). Those who endorsed one or both fake conditions provided less reliable data and were excluded from the analyses [18].

### Preference-based measures

The PROPr was estimated from the PROMIS-29 + 2 scale scores using the U.S. scoring function, and possible scores ranged from − 0.022 to 0.954 [6]. The PROMIS-29 + 2 comprises the PROMIS-29 plus a 2-item cognitive function scale [1]. The EQ-5D-5L preference score was estimated from the five EQ-5D-5L items using the U.S. scoring function, with a possible range of −0.573 to 1 [9]. Multi-attribute utility functions are used to estimate both the PROPr and EQ-5D-5L preference-based scores.

### NIH pain consortium research task force chronic pain definition

Using responses to the general health survey, we classified individuals as having chronic back pain based on the definition proposed by the NIH Pain Consortium Research Task Force: having pain that has persisted for at least 3 months and resulted in pain for at least half the days in the past six months [19].

### Pain impact measures

#### Oswestry disability index (ODI)

The ODI focuses on functional disability across a range of domains such as physical function, pain, and sleep. The 10 ODI items range from 0 to 5 and the total is scored on a 0−100 possible range with higher scores indicating worse disability [20].

#### Roland-Morris disability questionnaire (RMDQ)

The RMDQ asks about the impact of back pain on daily activities and yields an overall score that is a sum of 24 dichotomous items with a possible range of 0–24, with a higher score an indication of worse impact [21].

#### Pain intensity, interference with enjoyment of life, interference with general activity (PEG)

The PEG scale is a 3-item subset of the Brief Pain Inventory (BPI), and each item is administered using a 0 to 10 response scale with 10 indicating worse symptoms [22]. One PEG item is a BPI intensity item, and the other two are from the BPI interference scale. The PEG is scored as the average of the 3 items. The PEG was recommended by the U.S. National Pain Strategy and by the Surgeon General’s Turning the Tide campaign to reduce opioid use.

#### Subgroups for targeted treatment back screening tool (STarT Back)

The STarT Back screening tool queries the location of pain, functional impairment associated with back pain, and emotional well-being. The 9 STarT Back items are dichotomous (scored 0 or 1) with a total score ranging from 0 to 9 and higher scores indicating worse symptoms [23].

#### Graded chronic pain scale (GCPS)

The 7-item GCPS has a 3-item pain intensity score and a 3-item disability score [24]. The pain intensity scale assesses back pain at present, and average and worst pain in the past 6 months. The disability score reflects pain interference with daily activities, changed ability to do recreational, social, and family activities, and changed ability to work (including housework). Higher scores represent more pain and disability.

### Retrospective change items

Nine retrospective change items were included in the six-month follow-up sample: All items used “Compared to six months ago” at the beginning. Eight of the items followed with: (1) In general, how is your physical functioning now? (2) In general, how is your ability to participate in social roles and activities now? (3) In general, how is your pain now? (4) In general, how is your fatigue now? (5) In general, how is your mood? (6) In general, how is your thinking (also known as cognition)? (7) In general, how is your sleep now? (8) how would you rate your health in general now? These items were administered using five response options (*Much better now than six months ago*; *Somewhat better now than six months ago*; *About the same*; *Somewhat worse now than six months ago*; *Much worse now than six months ago*). One retrospective change item included different response options: Compared to six months ago, is your back pain problem… (*Much worse*; *A little worse*; *About the same*; *A little better*; *Moderately better; Much better; Completely gone*). We scored each of the nine items so that a higher score represented a more positive change in health.

### Subjects

Those who completed the baseline general health survey were 50% female, had a median age of 54 (range 18–94), 7% did not graduate from high school, 26% had a high school degree or general education diploma, 26% some college or AA degree, and 41% a bachelor’s degree or higher. Most of the sample was non-Hispanic White (70%), 12% were Hispanic, 10% were Black, 3% multi-racial, and 5% other. 59% were married, 20% were never married, 10% were divorced, 5% living with a partner, 5% were widowed, and 1% were separated. 44% were working full-time. The unweighted sample was similar in gender and education, slightly older (54 versus 48), and had fewer Hispanics (12% versus 17%) than the U.S. general population (2022 March Supplement of the Current Population Survey) [25]. Results were robust when post-stratification weights were used (not shown).

Those with back pain in the baseline sample were a little more likely than the overall sample to be female, older, less educated, White, never married, and less likely to work full-time. Specifically, the back pain subgroup was 52% female, had a median age of 57 (range 18–94), 7% did not graduate from high school, 29% had a high school degree or general education diploma, 29% had some college or AA degree, and 35% a bachelor’s degree or higher. Most of the sample was non-Hispanic White (73%), 10% were Hispanic, 8% were Black, 4% multi-racial, and 4% other. 60% were married, 16% were never married, 11% were divorced, 6% living with a partner, 6% were widowed, and 2% were separated. 37% were working full-time.

### Analysis plan

We report product-moment and intraclass correlations between the EQ-5D-5L and PROPr at the study baseline. Intraclass correlations can be estimated using either two-way mixed effects or random effects analysis of variance [26]. The mixed effects formula, with *N* representing the number of respondents and *MS*_*time*_ the mean square for the main effect of timepoint, is:

$${\left({\text{MS}}_{\text{between}} - {\text{MS}}_{\text{within}}\right)/{\text{MS}}_{\text{between}}}$$

*MS*_*between*_ is the mean square between respondents and *MS*_*within*_ is the mean square for the interaction of respondents and timepoint (test, retest). The random effects model is:

$${{\text{N}}\,\left({\text{MS}}_{\text{between}} - {\text{MS}}_{\text{within}}\right)/\left({\text{N}}\,{\text{MS}}_{\text{between}} + {\text{MS}}_{\text{time}} - {\text{MS}}_{\text{within}}\right)}$$

Rencz et al. [10] and Klapproth et al. [12] used the two-way random effects model based on absolute agreement to estimate the intraclass correlation between the EQ-5D-5L and PROPr. Qin et al. [27] noted that the two-way mixed effect ANOVA with interaction for absolute agreement is equivalent to the two-way random effects model. For completeness, we estimate the ICC using both two-way models.

We estimate product-moment correlations of demographic variables with the EQ-5D-5L and PROPr for the overall sample. Next, we compute baseline correlations between the preference and pain impact measures for those with back pain. We then estimate ordinary least squares (OLS) regression models with each preference measure as a dependent variable and the 22 medical conditions as independent variables. We hypothesized that more positive (better health) scores on the preference measures would be associated with less negative pain impact.

For those with back pain, we also estimate the product-moment correlation between change in the EQ-5D-5L and PROPr from baseline to six months later and correlations of the change in the two preference scores with the retrospective rating of change items. A correlation (r) of 0.100 corresponds to small, 0.243 medium, and 0.371 large based on Cohen’s [28] 0.2, 0.5, and 0.8 effect size (d) magnitude rules of thumb: r = $d\sqrt{{ d}_{}^{2}+4}$

Finally, we regressed the EQ-5D-5L score on the PROPr and vice versa. We used linear equating to address the problem of over-prediction of low scores and under-prediction of high scores due to regression to the mean [29]. We linearly transformed predicted scores from each regression model to have the same mean and SD as the observed EQ-5D-5L (PROPr) preference-based scores. We recoded scores that were outside of the observed range to the nearest minimum or maximum observed scores. OLS models were evaluated regarding adjusted R^2^ and estimated product-moment and intraclass correlations between the predicted and observed PROPr and EQ-5D-5L scores. In addition, we estimated the normalized mean absolute error (NMAE). In our implementation of the NMAE, we averaged deviations between observed and predicted scores by the standard deviation of the observed score. Low values of the NMAE indicate better performance. We also fit beta-binomial regression models for the preference-based scores to compare with the fit of the OLS models. Because beta-binomial models assume a 0–1 scale for utility, Khan and Morris [30] recoded EQ-5D-3L scores less than 0 to 0. When we did this, the beta-binomial regression models could not be estimated because quasi-Newton optimization did not improve the function value. Instead, we transformed utility values linearly to a 0–1 possible range: (observed value – minimum observed)/observed range.

## Results

### Correlation between EQ-5D-5L and PROPr in general population sample at baseline

The product-moment correlation between the EQ-5D-5L and PROPr preference scores was 0.69 at baseline. The two-way mixed ICC was 0.67, and the two-way random effects ICC was 0.34. The mean difference for the EQ-5D-5L and PROPr preference scores was 0.316, larger than their SDs: the EQ-5D-5L mean was 0.855 (SD = 0.195, score range: − 0.370 to 1.000) versus the PROPr mean of 0.539 (SD = 0.249, score range: − 0.018 to 0.954). 31% of the sample scored at the ceiling (highest possible score of 1) on the EQ-5D-5L.

### Correlations of EQ-5D-5L and PROPr with demographic variables (general population) and pain impact measures (back pain subgroup) at baseline

The EQ-5D-5L correlated significantly negatively with age, but the correlation between age and the PROPr was non-significant. The PROPr correlated more strongly than the EQ-5D-5L with female gender (Table 1). The EQ-5D-5L correlated slightly more strongly than the PROPr with all the pain impact measures (ODI, RMDQ, PEG, GCPS pain intensity score, GCPS disability score, and U.S. National Institutes of Health Pain Consortium Research Taskforce’s definition of chronic pain).

**Table 1:** Product-moment correlations of PROPr and EQ-5D.5L with demographic characteristics (*n* = 4098) and Pain Impact scales (*n* = 1528) at baseline

	PROPr	EQ-5D-5L
Age	0.03 (0.0891)	−0.08 (<0.0001)
Female gender	−0.11 (<0.0001)	−0.07 (<0.0001)
Education	0.19 (<0.0001)	0.19 (<0.0001)
Working full-time	0.18 (<0.0001)	0.22 (<0.001)
Pain impact measures		
Oswestry disability index	−0.69 (<0.0001).	−0.75 (<0.0001)
Roland-Morris disability questionnaire	−0.62 (<0.0001)	−0.65 (<0.0001)
Pain intensity, interference with enjoyment of life, interference with general activity (PEG) scale	−0.66 (<0.0001)	−0.73 (<0.0001)
StartBack	−0.66 (<0.0001)	−0.66 (<0.0001)
Graded Chronic pain scale pain intensity score	−0.55 (<0.0001)	−0.58 (<0.0001)
Graded Chronic pain scale disability score	−0.65 (<0.0001)	−0.68 (<0.0001)
United States national institutes of health pain consortium research taskforce’s definition of chronic pain	−0.30 (<0.0001)	−0.31 (<0.0001)

*Note* Higher scores are worse for the seven pain impact measures

### Associations of EQ-5D-5L and PROPr with health conditions in the general population sample at baseline

OLS regression of the EQ-5D-5L on the 22 conditions yielded an adjusted R^2^ of 39%, while the PROPr R^2^ was 41% (Table 2). Fifteen of the 22 conditions were significantly associated with the EQ-5D-5L: depression; sciatica; COPD; trouble sleeping; stroke; back pain; trouble seeing; arthritis; anxiety; stomach trouble; diabetes, dermatitis; hypertension; neck pain; and allergies (suppression effect). The largest regression coefficients were observed for depression (−0.078), followed by sciatica (−0.077). Thirteen of the 22 conditions were significantly associated with PROPr trouble sleeping, depression, back pain, trouble seeing, diabetes, sciatica, anxiety, COPD, dermatitis, stomach trouble, arthritis, neck pain, and high cholesterol (suppression effect). The largest regression coefficients were for trouble sleeping (−0.152), followed by depression (−0.112).

**Table 2:** Associations of chronic conditions with PROPr and EQ-5D-5L in the general population sample (*n* = 4098): regression coefficients (zero-order correlations)

Condition (% of sample)	PROPr (*R*^2^ = 0.41)	EQ-5D-5L (*R*^2^ = 0.39)
Hypertension (38%)	−0.009 (−0.16)	−0.024**** (−0.20)
High cholesterol (38%)	0.015* (−0.11)	0.001 (−0.13)
Heart disease (6%)	−0.001 (−0.07)	−0.004 (−0.09)
Angina (2%)	−0.022 (−0.09)	−0.025 (−0.10)
Heart attack (3%)	−0.001 (−0.06)	−0.001 (−0.07)
Stroke (3%)	−0.030 (−0.08)	−0.058*** (−0.11)
Asthma (13%)	−0.013 (−0.14)	−0.009 (−0.13)
Cancer (10%)	0.020 (−0.03)	0.009 (−0.05)
Diabetes (13%)	−0.057**** (−0.16)	−0.032**** (−0.17)
Chronic obstructive pulmonary disease (5%)	−0.044** (−0.15)	−0.069**** (−0.19)
Arthritis (30%)	−0.031**** (−0.26)	−0.046**** (−0.32)
Anxiety (20%)	−0.055* (−0.35)	−0.041**** (−0.34)
Depression (20%)	−0.112**** (−0.46)	−0.078**** (−0.38)
Allergies (45%)	0.012 (−0.15)	0.015** (−0.14)
Back pain (38%)	−0.069**** (−0.37)	−0.052**** (−0.38)
Sciatica (17%)	−0.057**** (−0.31)	−0.077**** (−0.36)
Neck pain (20%)	−0.029*** (−0.29)	−0.025*** (−0.30)
Trouble seeing (15%)	−0.061**** (−0.29)	−0.047**** (−0.27)
Dermatitis (10%)	−0.044**** (−0.16)	−0.030*** (−0.16)
Stomach trouble (15%)	−0.039**** (−0.29)	−0.039**** (−0.29)
Trouble hearing (15%)	−0.014 (−0.15)	−0.009 (−0.16)
Trouble sleeping (15%)	−0.152**** (−0.46)	−0.065**** (−0.36)
Intercept	0.692	0.974

*p < 0.05; **p < 0.01; ****p < 0.001; ****p < 0.0001

*Note* Product-moment correlation = 0.86 between regression coefficients

**Fig. 1:**
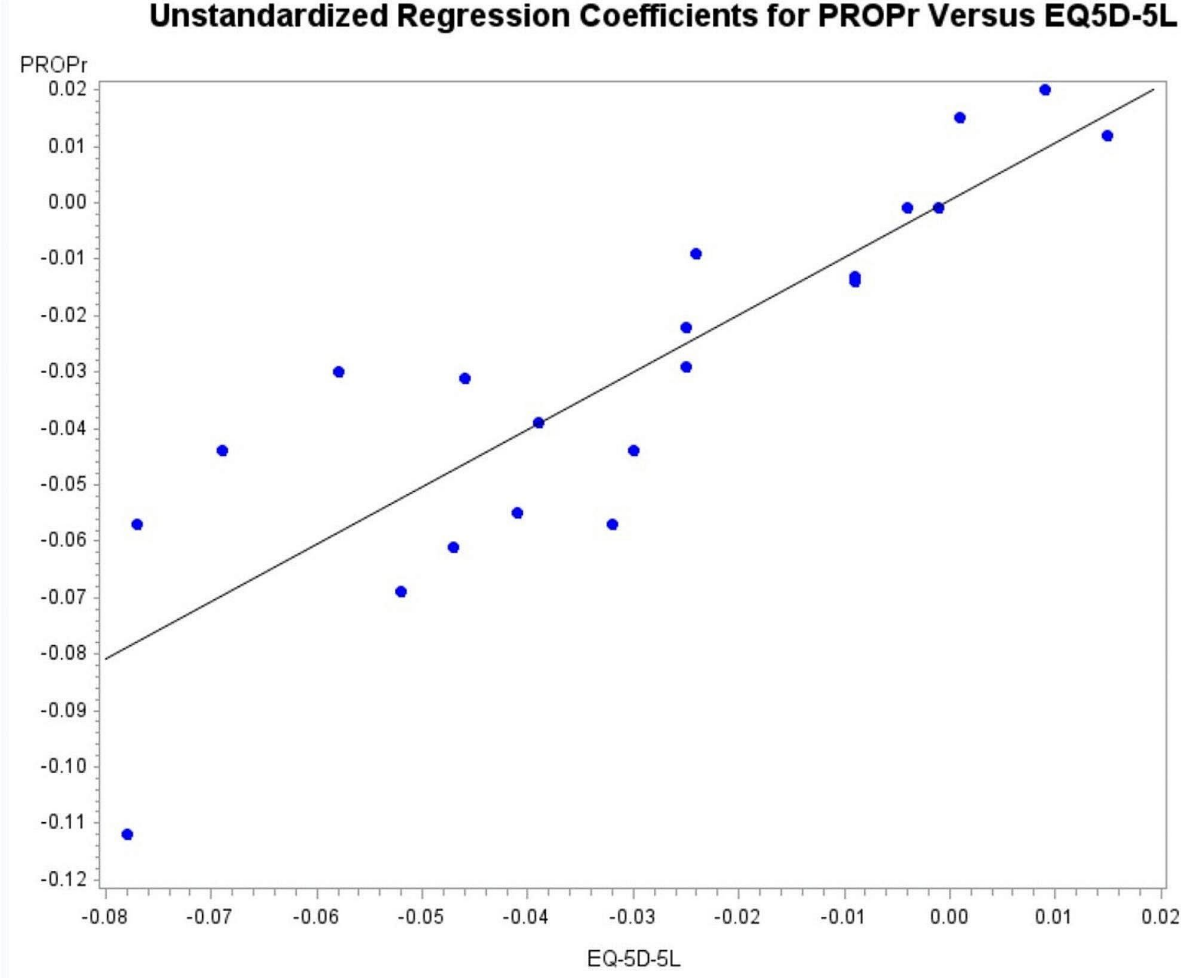
Unstandardized health condition regression coefficients for PROPr and EQ-5D-5L

The product-moment correlation between the 22 betas for the PROPr and the EQ-5D-5L was 0.86, indicating similar patterns of unique associations (see Fig. 1).

### Correlations of six month change in EQ-5D-5L and PROPr with retrospective change items for back pain subsample

The mean change in the PROPr and EQ-5D-5L preference scores from baseline to six months later was 0.00. The product-moment correlation between change in the two measures was 0.34. Table 3 provides product-moment correlations between change in these measures and the nine retrospective measures of change administered as part of the 6-month survey. The correlations were small (less than 0.243).

**Table 3:** Product-moment correlations of change from baseline to six months later in PROPr and EQ-5D-5L with retrospective change in back pain subsample (*n* = 1250)

Retrospective item	Change in PROPr	Change in EQ-5D-5L
Physical function (65%)	0.13 (*p* < 0.0001)	0.19 (*p* < 0.0001)
Social (76%)	0.15 (*p* < 0.0001)	0.15 (*p* < 0.0001)
Pain (62%)	0.15 (*p* < 0.0001)	0.20 (*p* < 0.0001)
Fatigue (65%)	0.14 (*p* < 0.0001)	0.13 (*p* < 0.0001)
Mood (66%)	0.10 (*p* = 0.0003)	0.11 (*p* = 0.0002)
Cognition (76%)	0.06 (*p* = 0.0317)	0.09 (*p* = 0.0016)
Sleep (66%)	0.14 (*p* < 0.0001)	0.10 (*p* < 0.0001)
Health (64%)	0.12 (*p* < 0.0001)	0.20 (*p* < 0.0001)
Back pain (58%)	0.14 (*p* < 0.0001)	0.16 (*p* < 0.0001)

*Note* PROPr = PROMIS-29 + 2 preference score; EQ-5D-5L = EuroQOL preference score. Percentages in parentheses indicate those who reported they were the same on the retrospective change item

### Predicting EQ-5D-5L from PROPr in general population sample at baseline

The adjusted R^2^ for the OLS regression of the EQ-5D-5L on the PROPr was 48%. Adding age and gender to the model only improved the adjusted R^2^ to 49% so these variables were not used in mapping. The linearly equated EQ-5D-5L had a mean of 0.830 and an SD of 0.165 compared with the observed EQ-5D-5L mean of 0.855 and SD of 0.195. The NMAE was 0.47. The equated EQ-5D-5L preference scores correlated (product-moment) 0.72 (*n* = 4092; *p* < 0.0001) with the observed EQ-5D-5L preference scores, and the intra-class correlation (two-way random effects model) between equated and observed EQ-5D-5L preference scores was 0.71. The equations to predict the EQ-5D-5L are as follows:

$${\text{EQ-5D-5Lpredicted }} = 0.563{\text{ }} + {\text{ }}0.543{\text{ }}*{\text{ PROPr}}$$

$$\eqalign{&{\text{EQ-5D-5L\_equated }} = {\text{ }}0.855{\text{ }} + {\text{ }}\left( {0.195/0.135} \right) \cr &\quad\quad* \left( {{\text{EQ-5D-5Lpredicted }} - {\text{ }}0.855} \right)\cr}$$

$$\eqalign{&{\text{If}}\,{\text{EQ-5D-5L\_equated}} < - 0.573\,{\text{then}} \cr &\quad\,{\text{EQ-5D-5L\_equated }} = {\text{ }} -0.573 \cr}$$

$$\eqalign{&{\text{Else}}\,{\text{if}}\,{\text{EQ-5D-5L\_equated}}\, > \,1\,{\text{then}} \cr &\quad{\text{EQ-5D-5L\_equated}} = {\text{ }}1 \cr}$$

The beta-binomial regression model was a slight improvement over OLS regression. The NMAE was 0.41, the product-moment correlation between predicted and observed EQ-5D-5L was 0.78, and the intra-class correlation was 0.75.

### Predicting PROPr from EQ-5D-5L in general population sample at baseline

The adjusted R^2^ in the OLS regression of the PROPr on the EQ-5D-5L was 48%. Adding age and gender to the model only improved the adjusted R^2^ to 49%, and the gender coefficient was not significant (*p* =.4988), so these variables were not used in the mapping. The equated PROPr had a mean of 0.551 and an SD of 0.205 compared with the observed PROPr mean of 0.538 and SD of 0.249. The NMAE was 0.54. The equated PROPr preference scores correlated (product-moment) 0.73 (*n* = 4092; *p* <.0001) with the observed PROPr preference scores, and the intra-class correlation (two-way random effects model) between equated and observed PROPr preference scores was 0.71. The OLS equations to predict the PROPr are as follows:

$${\text{PROPrpredicted}}\, = \, - 0.218 + 0.885\,*\,{\text{EQ-5D-5L}}$$

$$\eqalign{&{\text{PROPr\_equated}}\, = \,0.538 + \left( {0.249/0.173} \right) \cr & \quad\quad *\,\left( {{\text{PROPrpredicted}}\, - \,0.538} \right) \cr}$$

$$\eqalign{&{\text{If}}\,{\text{PROPr\_equated}}\, < {\text{ }} - 0.022 \,{\text{then}}\,\cr &\quad{\text{PROPr\_equated}} = -0.022 \cr}$$

$$\eqalign{&{\text{Else}}\,{\text{if}}\,{\text{PROPr\_equated}}\, > \,1\,{\text{then}}\,\cr & \quad {\text{PROPr\_equated}} = 1 \cr}$$

The beta-binomial regression prediction was equivalent to the OLS model. The NMAE was 0.54, the product-moment correlation between predicted and observed EQ-5D-5L was 0.73, and the intra-class correlation was 0.70.

## Discussion

The current study compared the EQ-5D-5L and PROPr in a U.S. sample. The lower PROPr mean score than the EQ-5D-5L and the correlation between the PROPr and EQ-5D-5L (*r* =.69 and two-way random effects ICC = 0.34) were like those reported by others in cross-sectional analyses [10–12]. In addition, the stronger correlation of the EQ-5D-5L with age is consistent with what was found by Rencz et al. [10]. However, similar age trends for the two measures were observed in a study of German inpatients with rheumatological and psychosomatic conditions [12]. The EQ-5D-5L preference score has had inconsistent associations with gender in prior studies [31] and had a smaller negative correlation with being female than the PROPr in the current study. The correlations of the preference measures with the pain impact measures among those with back pain were similar but slightly larger for the EQ-5D-5L. This is consistent with the fact that in terms of score impact, pain/discomfort is very influential for the EQ-5D-5L score [9].

The 22 conditions accounted for similar amounts of variance in the EQ-5D-5L and PROPr preference scores at baseline (39% and 41%, respectively). This study’s correlation between the regression coefficients for the EQ-5D-5L and PROPr of 0.86 is in the ballpark of what Hanmer et al. [11] reported (i.e., > 0.70). The correlations of change in the EQ-5D-5L and PROPr from baseline to six months later with the nine retrospective change items were small.

The OLS regression model indicated a 48% shared variance between the EQ-5D-5L and PROPr. The intraclass correlations for equated preference scores (0.70 and 0.72) are good [32], especially considering test-retest correlations of 0.77 for the EQ-5D-5L [33]. The NMAE of 0.47 (predicting EQ-5D-5L) and 0.54 (predicting PROPr) indicate that, on average, the predicted values were within a half-standard deviation of the observed scores.

Using a well-known probability-based panel representative of the U.S. population strengthens the study. However, the survey was administered only in English, and the longitudinal sample was limited to those with back pain. Most reported no change on the retrospective change items (from 58% for change in back pain to 76% for ability to participate in social roles and activities and cognitive function). The study used self-report data, and information about health conditions documented by physicians was not collected. The study was also limited to the HRQOL measures examined. The PROPr score was derived from the PROMIS-29 + 2, and the EQ-5D-5L includes only five questions. In addition, the sample was limited to adults in the U.S. who may not have represented other countries. Some mapping studies include gender and age to improve prediction [34]. We showed that including age and gender in the regression models increased adjusted R^2^ by only 1% point. Finally, the estimated scores should be limited to group-level applications because of the lack of accuracy of individual-level estimates.

While the dependent variables were skewed, estimates of the regression line are generally robust to the assumption that errors are normally distributed and support tests of means [35]. Moreover, we used linear equating to address the problem of over-predicting at the lower and underpredicting at the upper end. Methods other than OLS have been used, such as Tobit and Censored Least Absolute Deviation, mixture models, and adjusted limited dependent variable mixture models. Beta-binomial regression was found to perform better than OLS for several fit criteria (root mean squared error, mean absolute error, normal root mean squared error, normalized mean absolute error, and correlation between predicted and observed values) in a prior study, but the fit was very similar to two decimal places (e.g., root mean squared error of 0.122 versus 0.119 for OLS and beta-binomial, respectively) [36]. Beta-binomial regression in this study yielded a slightly better prediction than OLS regression for the EQ-5D-5L and a similar prediction for the PROPr. Because of the similarity of fit between the OLS and beta-binomial models and complication in the latter due to the need to transform the estimated utility values to the 0–1 possible range, we provide the OLS regression equations for use in cost-effectiveness research and meta-analyses.

The results of this study and prior work indicate that the EQ-5D-5L and PROPr preference scores are substantially associated cross-sectionally (*r* =.69), falling within the 0.61–0.71 range of correlations found among the EQ-5D-3L, HUI-2/3, QWB-SA, and SF-6D in a U.S. national survey sample of 3844 adults [37]. In addition, the EQ-5D-5L and PROPr had similar associations with other variables in the current study.

## Conclusion

The OLS regression equations from one preference measure to another can facilitate cost-effectiveness research and meta-analyses. Future studies are needed to compare the EQ-5D-5L and PROPr for different health conditions and interventions to provide additional information on the relative validity of these two measures. Additional longitudinal evaluation of the two measures and comparison of the PROPr with other preference-based measures would also be valuable.

## Data Availability

The data are available at https://www.openicpsr.org/openicpsr/project/198049/version/V1/view.
